# A multi-sequence and habitat-based MRI radiomics signature for preoperative prediction of MGMT promoter methylation in astrocytomas with prognostic implication

**DOI:** 10.1007/s00330-018-5575-z

**Published:** 2018-07-23

**Authors:** Jingwei Wei, Guoqiang Yang, Xiaohan Hao, Dongsheng Gu, Yan Tan, Xiaochun Wang, Di Dong, Shuaitong Zhang, Le Wang, Hui Zhang, Jie Tian

**Affiliations:** 1 Key Laboratory of Molecular Imaging, Institute of Automation, Chinese Academy of Sciences, Beijing, 100190 China; 2 Beijing Key Laboratory of Molecular Imaging, Beijing, 100190 China; 3 University of Chinese Academy of Sciences, Beijing, 100049 China; 4 Department of Radiology, First Clinical Medical College, Shanxi Medical University, Taiyuan, 030001 Shanxi Province China

**Keywords:** Astrocytoma, Methylation, Prognosis, Diagnostic imaging, ROC curve

## Abstract

**Objectives:**

Oxygen 6-methylguanine-DNA methyltransferase (MGMT) promoter methylation is a significant prognostic biomarker in astrocytomas, especially for temozolomide (TMZ) chemotherapy. This study aimed to preoperatively predict MGMT methylation status based on magnetic resonance imaging (MRI) radiomics and validate its value for evaluation of TMZ chemotherapy effect.

**Methods:**

We retrospectively reviewed a cohort of 105 patients with grade II-IV astrocytomas. Radiomic features were extracted from the tumour and peritumoral oedema habitats on contrast-enhanced T1-weighted images, T2-weighted fluid-attenuated inversion recovery images and apparent diffusion coefficient (ADC) maps. The following radiomics analysis was structured in three phases: feature reduction, signature construction and discrimination statistics. A fusion radiomics signature was finally developed using logistic regression modelling. Predictive performance was compared between the radiomics signature, previously reported clinical factors and ADC parameters. Validation was additionally performed on a time-independent cohort (n = 31). The prognostic value of the signature on overall survival for TMZ chemotherapy was explored using Kaplan Meier estimation.

**Results:**

The fusion radiomics signature exhibited supreme power for predicting MGMT promoter methylation, with area under the curve values of 0.925 in the training cohort and 0.902 in the validation cohort. Performance of the radiomics signature surpassed that of clinical factors and ADC parameters. Moreover, the radiomics approach successfully divided patients into high-risk and low-risk groups for overall survival after TMZ chemotherapy (*p* = 0.03).

**Conclusions:**

The proposed radiomics signature accurately predicted MGMT promoter methylation in patients with astrocytomas, and achieved survival stratification for TMZ chemotherapy, thus providing a preoperative basis for individualised treatment planning.

**Key Points:**

*• Radiomics using magnetic resonance imaging can preoperatively perform satisfactory prediction of MGMT methylation in grade II-IV astrocytomas.*

*• Habitat-based radiomics can improve efficacy in predicting MGMT methylation status.*

*• Multi-sequence radiomics signature has the power to evaluate TMZ chemotherapy effect.*

**Electronic supplementary material:**

The online version of this article (10.1007/s00330-018-5575-z) contains supplementary material, which is available to authorized users.

## Introduction

Astrocytoma is the most common type of glioma, and carries a poor prognosis [1, 2]. The average survival time ranges from 17 weeks to 3 years [2, 3]. Fortunately, a subgroup of grade II-IV astrocytoma patients with oxygen 6-methylguanine-DNA methyltransferase (MGMT) promoter methylation show good responses to temozolomide (TMZ) chemotherapy and improved survival after treatment, which underscored the role of MGMT as a judicious molecular biomarker with a prognostic implication [4–7]. Preoperative identification of MGMT promoter methylation would be of great clinical significance in selecting potential patients benefiting from TMZ chemotherapy, thus assisting with planning the therapy regime. However, the standard approach for MGMT status confirmation requires a large tissue sample that is usually obtained through surgery. For patients with unresectable tumours, biopsy runs the risk of neurological deficits and can accordingly jeopardise the quality of life of the patient [8, 9]. Thus, there is an urgent need in clinical practice for preoperative and non-invasive prediction of MGMT promoter methylation in grade II-IV astrocytomas.

Magnetic resonance imaging (MRI), as a powerful non-invasive diagnostic imaging tool for astrocytoma management [10], opens up the possibility of having this preoperative prediction. Previous studies have verified that certain radiological characteristics on MR images such as tumour necrosis, enhancement patterns and tumour location are associated with MGMT promoter methylation [11, 12]. However, subjective judgements by radiologists are vulnerable to inter-observer variability and generally lack power and accuracy. In contrast, a newly emerging technology termed radiomics could resolve this problem by quantitative imaging analysis. Radiomics converts encrypted medical images into usable data by extracting high-throughput imaging features and relating imaging feature data to targeted clinical outcomes [13, 14]. Regions of interest (ROIs) are delineated on the tumour and sub-regions of the lesion known as habitats. Thus, radiomics not only offers holistic imaging information, but also explores the microenvironment of the tumour by analysing explicit sub-regional features that describe genetic heterogeneity more granularly [15]. For gliomas, radiomics studies on molecular subtype prediction have demonstrated sufficient predictive accuracy for isocitrate dehydrogenase (IDH) and 1p19q codeletion [16, 17]. Habitat-based radiomics have also been shown to have the capability to identify survival stratification in glioblastomas (GBMs) [18]. These studies suggest that habitat-based radiomics may be similarly useful for the preoperative prediction of MGMT promoter methylation in patients with astrocytomas.

In this study, we investigated the utility of a multi-sequence and multi-habitat MR radiomics signature as a preoperative and non-invasive biomarker of MGMT methylation prediction in patients with grade II–IV astrocytomas, and discuss the prognostic implications for survival stratification on TMZ chemotherapy response.

## Materials and methods

### Patients

This retrospective study was approved by the institutional review board. We reviewed 105 patients who were newly diagnosed with grade II–IV astrocytoma from October 2011 to March 2017. The inclusion and exclusion criteria are given in the Online Supplemental Appendix E1. The patient recruitment pathway is shown in Online Supplemental Fig. 1.

A total of 105 patients met the study criteria and were divided into a training dataset (31 December 2011 to 2 November 2015, n = 74) and a time-independent validation dataset (16 November 2015 to 21 March 2017, n = 31). Demographic and clinical data were acquired from medical reports, including sex, age, grade and radiological characteristics (Table 1).

**Table 1:** Clinical characteristics in the training and validation cohorts

Characteristic	Training cohort N=74			Validation cohort N=31			*p* (Inter)
	MGMT (+)	MGMT (-)	*p* (Intra)	MGMT (+)	MGMT (-)	*p* (Intra)	
Gender			0.674			0.139	0.902
Male	32 (43.2)	10 (13.5)		8 (25.8)	10 (32.3)		
Female	23 (31.1)	9 (12.2)		10 (32.3)	3 (9.7)		
Age, y			0.225			0.701	0.195
≤52	32 (43.2)	8 (10.8)		13 (41.9)	8 (25.8)		
>52	23 (31.1)	11 (14.9)		5 (16.1)	5 (16.1)		
Grade			0.258			0.509	0.905
II	24 (32.4)	5 (6.8)		7 (22.6)	4 (12.9)		
III	22 (29.7)	8 (10.8)		9 (29.0)	5 (16.1)		
IV	9 (12.2)	6 (8.1)		2 (6.5)	4 (12.9)		
Tumour size			0.508			1.000	0.073
≤6cm	27 (36.5)	11 (14.9)		6 (19.4)	4 (12.9)		
>6cm	28 (37.8)	8 (10.8)		12 (38.7)	9 (29.0)		
Tumour centre location			0.350			1.000	0.980
Left hemisphere	25 (33.8)	11 (14.9)		9 (29.0)	6 (19.4)		
Right hemisphere	30 (40.5)	8 (10.8)		9 (29.0)	7 (22.6)		
Frontal lobe			0.674			0.013^*^	0.902
Yes	32 (43.2)	10 (13.5)		14 (45.2)	4 (12.9)		
No	23 (31.1)	9 (12.2)		4 (12.9)	9 (29.0)		
Occipital lobe			0.702			0.497	0.742
Yes	5 (6.8)	3 (4.1)		2 (6.5)	0 (0.0)		
No	50 (67.6)	16 (21.6)		16 (51.6)	13 (41.9)		
Parietal lobe			0.264			0.023^*^	0.116
Yes	13 (17.6)	7 (9.5)		0 (0.0)	4 (12.9)		
No	42 (56.8)	12 (16.2)		18 (58.1)	9 (29.0)		
Temporal lobe			0.706			0.710	0.728
Yes	35 (47.3)	6 (8.1)		6 (19.4)	6 (19.4)		
No	20 (27.0)	13 (17.6)		12 (38.7)	7 (22.6)		
Insular lobe			0.140			0.625	0.817
Yes	9 (12.2)	0 (0.0)		2 (6.5)	3 (9.7)		
No	46 (62.2)	19 (25.7)		16 (51.6)	10 (32.3)		
Involving cortex matter			0.635			1.000	0.742
Yes	48 (64.9)	18 (24.3)		17 (54.8)	12 (38.7)		
No	7 (9.5)	1 (1.4)		1 (3.2)	1 (3.2)		
Involving deep white matter			0.910			0.497	0.238
Yes	46 (62.2)	15 (20.3)		16 (51.6)	13 (41.9)		
No	9 (12.2)	4 (5.4)		2 (6.5)	0 (0.0)		
Involving pial matter			1.000			1.000	1.000
Yes	48 (64.9)	16 (21.6)		16 (51.6)	11 (35.5)		
No	7 (9.5)	3 (4.1)		2 (6.5)	2 (6.5)		
Involving ependymal membrane			0.555			1.000	0.600
Yes	19 (25.7)	8 (10.8)		8 (25.8)	5 (16.1)		
No	36 (48.6)	11 (14.9)		10 (32.3)	8 (25.8)		
Tumour cross midline			0.949			1.000	0.735
Yes	11 (14.9)	3 (4.1)		3 (9.7)	2 (6.5)		
No	44 (59.5)	16 (21.6)		15 (48.4)	11 (35.5)		
Oedema cross midline			1.000			1.000	0.416
Yes	12 (16.2)	4 (5.4)		5 (16.1)	4 (12.9)		
No	43 (58.1)	15 (20.3)		13 (41.9)	9 (29.0)		
Border			0.171			0.099	0.455
Well-defined	14 (55.4)	8 (10.8)		2 (6.5)	5 (16.1)		
Ill-defined	41 (18.9)	11 (14.9)		16 (51.6)	8 (25.8)		
Haemorrhage			0.254			1.000	0.134
Yes	11 (14.9)	1 (1.4)		5 (16.1)	4 (12.9)		
No	44 (59.5)	18 (24.3)		13 (41.9)	9 (29.0)		
Cystic and necrosis			0.751			1	0.053
No	15 (20.3)	5 (6.8)		7 (22.6)	5 (16.1)		
≤25%	21 (28.4)	5 (6.8)		4 (12.9)	3 (9.7)		
25%-50%	11 (14.9)	5 (6.8)		1 (3.2)	1 (3.2)		
≥50%	8 (10.8)	4 (5.4)		6 (19.4)	4 (12.9)		
Oedema degree			0.011^*^			0.275	0.563
≤1.6	33 (44.6)	5 (6.8)		10 (32.3)	4 (12.9)		
>1.6	22 (29.7)	14 (18.9)		8 (25.8)	9 (29.0)		
Enhancement style			0.010^*^			0.034^*^	0369
No	15 (20.3)	3 (4.1)		4 (19.4)	0 (0.0)		
Ring enhancement	20 (27.0)	15 (20.3)		8 (25.8)	8 (25.8)		
Nodular enhancement	11 (14.9)	0 (0.0)		6 (19.4)	2 (6.5)		
Irregular reinforcement	9 (12.2)	1 (1.4)		0 (0.0)	3 (9.7)		
Enhancement degree			0.287			0.211	0.401
No	15 (20.3)	3 (4.1)		4 (12.9)	0 (0.0)		
Slight	5 (6.8)	0 (0.0)		2 (6.5)	1 (3.2)		
Obvious	35 (47.3)	16 (21.6)		12 (38.7)	12 (38.7)		
Signal characteristics			1.000			0.497	0.904
Homogeneous	5 (6.8)	2 (2.7)		2 (6.5)	0 (0.0)		
Heterogeneous	50 (67.6)	17 (23.0)		16 (51.6)	13 (41.9)		

*MGMT(+)* patients with oxygen 6-methylguanine-DNA methyltransferase (MGMT) methylation, *MGMT(-)* patients without MGMT methylation, *p (Intra)* the result of uni-variable analyses between methylated and unmethylated groups, *p(Inter)* significant difference between training and validation cohorts

Unless otherwise specified, data are numbers of patients, with percentages in parentheses

^*^p < 0.05

For evaluation of the TMZ chemotherapy effect, the inclusion criteria were further expanded in the 105 patients: (1) patients receiving adjuvant treatment following surgery consisting of either (a) concomitant radiation plus TMZ followed by adjuvant TMZ or (b) TMZ; (2) overall survival (OS) was categorised according to death or survival exceeding the median survival of patients with each tumour grade (605 days for grade II, 398 days for grade III and 169 days for grade IV). Twenty-two patients met the inclusion criteria for the TMZ survival analysis. Demographic and clinical data of the 22 patients are shown in Table 2.

**Table 2:** Clinical characteristics and MGMT predicted outcome for patients with TMZ chemotherapy

Characteristic	Patients N=22
Gender	
Male	13 (59.1)
Female	9 (40.9)
Age, y	
≤49	12 (54.5)
>49	10 (45.5)
Grade	
II	6 (27.3)
III	11 (50.0)
IV	5 (22.7)
MGMT	
+	17 (77.2)
–	5 (22.8)
Predicted MGMT	
MGMT(+)	14 (82.3)
MGMT(-)	3 (17.7)

*MGMT(+)* patients with oxygen 6-methylguanine-DNA methyltransferase (MGMT) methylation, *MGMT(-)* patients without MGMT methylation

Unless otherwise specified, data are numbers of patients, with percentages in parentheses

The average age was 49 years, thus we divided patients into an age ≤ 49 years group and an age > 49 years group

### MGMT methylation testing

The methylation status of the MGMT promoter was assessed using pyrosequencing analysis as described elsewhere [19]. Briefly, each tumour specimen was histologically investigated by macro-dissection to guarantee a tumour cell content of at least 80%. DNA was extracted using the Simlex OUP ® FFPE DNA extraction kit (TIB, China) and quantified by spectrophotometry using a NanoDrop 2000 (Thermo Fisher Scientific, Loughborough, UK). Bisulphite modification of the extracted DNA was performed using the BisulFlash™ DNA modification kit (EpiGentek, Farmingdale, NY, USA). The PCR amplification and conditions are given in Online Supplemental Appendix E2.

### MRI acquisition

Preoperative MRI was performed with a 3.0-T scanner (Signa HDxt, GE Healthcare, USA) using an 8-channel array coil. The acquisition protocols for CE-T1-WI, T2-FLAIR and DWI are in given in Online Supplemental Appendix E3.

### Demographic and clinical characteristic analysis

Differences between the training and validation cohorts and between the intra- MGMT methylated and unmethylated groups in terms of demographic and clinical factors were assessed with Pearson’s chi-square tests or Fisher’s exact tests for categorical variables and Student’s t-tests or Mann-Whitney U tests for continuous variables.

### Sample size and power calculation

According to the thumb rule, the sample size needed to cover 10–15 observations per predictor variable to yield a stable estimate [20]. In our study, the maximum number of included features for radiomics signature construction was 5 (T2-FLAIR sequence on tumour area). Thus, the training dataset needed to include at least 50 patients. For the validation dataset power calculation, a sample of > 11 patients was required to provide 80% power and a type I error rate of 5% [21]. Our study cohort included 105 patients with 74 in the training dataset and 31 in the validation dataset, which met the sample size requirement.

### Process of radiomics analysis

**Fig. 1:**
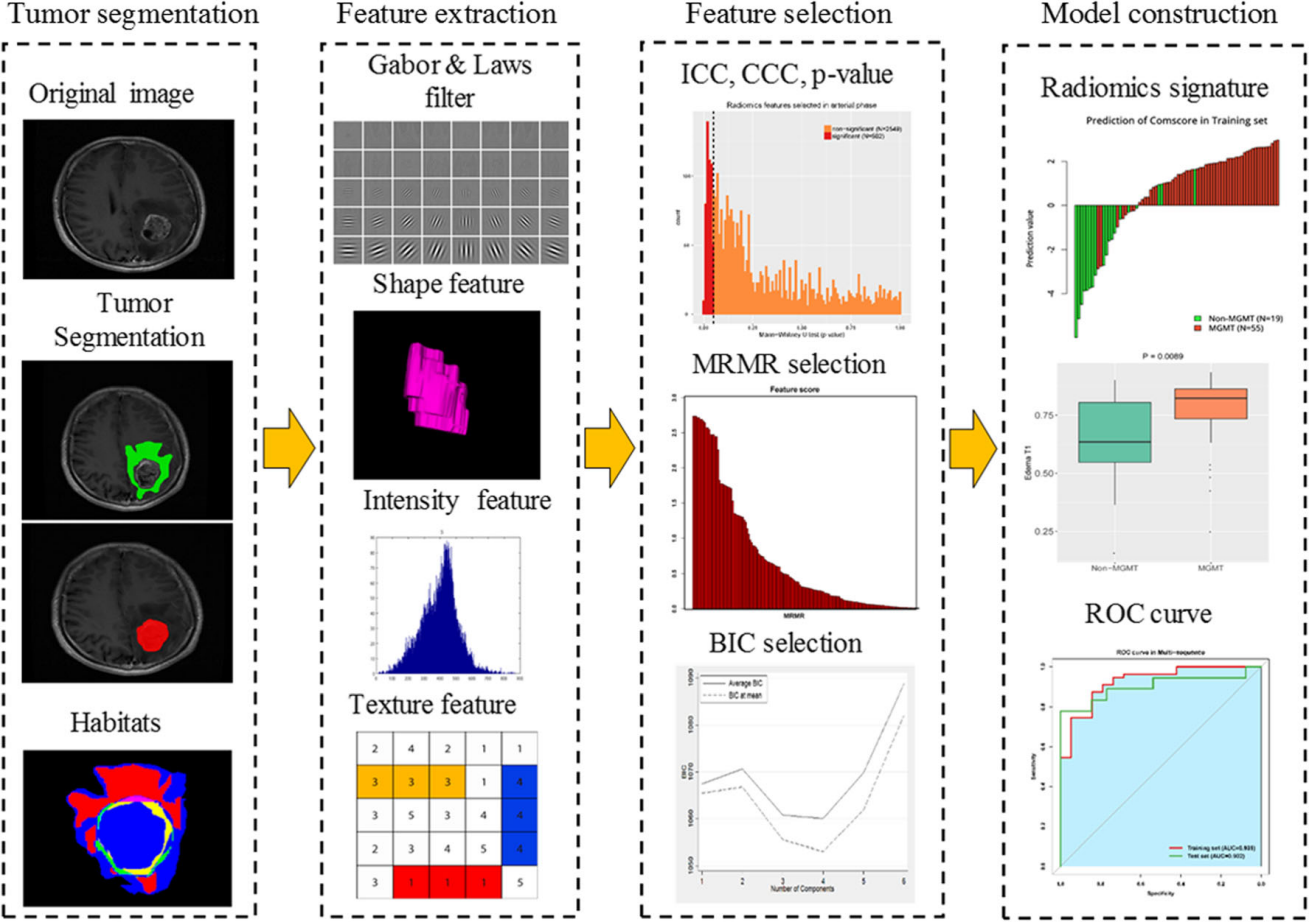
Radiomics workflow. The radiomics process included four parts: region of interest (ROI) segmentation on each habitat and sequence, feature extraction, feature selection and model construction. ROI was delineated on both the tumour and the peritumoral habitats on contrast-enhanced T1-weighted images and T2-FLAIR images. On each ROI, a set of 3,051 features were extracted. We performed fundamental stability and reproducibility analysis before using maximum relevance and minimum redundancy algorithm. The radiomics signature was generated by logistic regression with Bayesian information criteria as the stopping rule. Further performance evaluation was explored including receiver operating characteristics, decision curve analysis and survival stratification

**Fig. 2:**
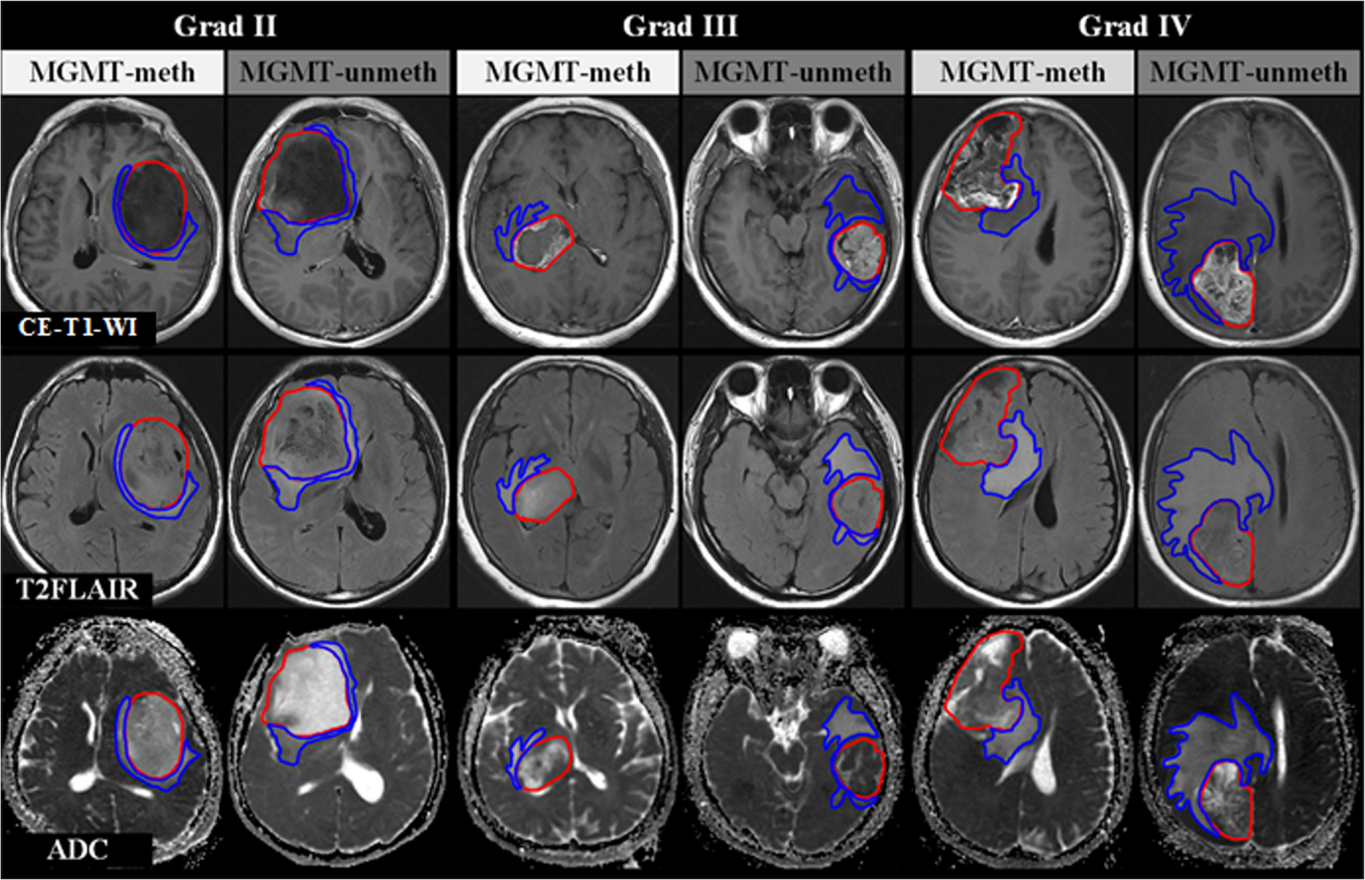
Tumour and oedema area segmentations are shown by the red and green lines, respectively. Oedema degree was obvious in the MGMT unmethylated group compared to the MGMT methylated group, especially for higher-grade astrocytomas (III and IV)

The radiomics analysis was structured into four parts: ROI segmentation, feature extraction, feature selection and model construction (Fig. 1). In brief, we performed a manual ROI with overlapped area by two radiologists (10 and 15 years of experience, respectively) on tumour and oedema habitats from T1-WI, T2-FLAIR and ADC maps, and the two radiologists were blinded to the final diagnosis and the MGMT methylation status. Examples of six typical segmentation cases according to grade and MGMT methylation status are shown in Fig. 2. The median ROI of each habitat and sequence is shown in Online Supplemental Table 3. A set of 3,051 imaging features was extracted including textural and non-textural features. Feature stability and reproducibility was estimated by Intra-class correlation coefficients and concordance correlation coefficients. Further feature selection was performed based on minimum redundancy and maximum relevance algorithm. Single radiomics signatures from each sequence and habitat, and a fusion radiomics signature were finally constructed using logistic regression modelling. A detailed description is given in Online Supplemental Appendix E4.

### Clinical and radiological factors for MGMT prediction

#### Clinical factor analysis and ADC parameter calculation

A univariate analysis was initially applied to select useful clinical factors (*p* < 0.1). Then, a forward selection (likelihood ratio) multi-variable analysis was performed to select clinical factors with *p* < 0.05. Additionally, we introduced ADC parameters (mean tumour ADC values and mean peritumoral oedema ADC values) associated with MGMT promoter methylation as reported in the literature [22]. Tumour and oedema ADC values were additionally integrated as a clinical model by logistic regression based on the training cohort.

#### Combined model with radiomics signature, clinical factor, and ADC values

To achieve a holistic information-gathered network, we generated a comprehensive model including the fusion radiomics signature, the selected clinical factor (oedema degree), and two ADC values (the tumour and oedema areas). Considering a correlation between the radiomics signature and other factors as well as the model complexity, we adopted the Akaike information criterion (AIC) to select optimal incorporated factors and used logistic regression modelling to perform model construction.

### Performance evaluation

Receiver operating characteristic (ROC) curves were plotted and area under the curve (AUC), specificity and sensitivity were calculated for the fusion radiomics signature, selected clinical factor and ADC parameter. We further performed stratification analysis for the fusion radiomics signature by grouping the cohorts according to age, gender and grade. We chose decision curve analysis (DCA) to estimate the clinical usefulness of the developed fusion radiomics signature and used the Delong test to explore whether the fusion radiomics signature performed better than the traditional clinical model and ADC parameter.

### Prognostic value analysis

A Kaplan-Meier curve was plotted based on the fusion radiomics signature in order to stratify the OS in patients treated with adjuvant TMZ chemotherapy. The log-rank test was used to determine whether there were statistical differences between the two survival groups.

### Statistical analysis

We performed the statistical analysis with PASW Statistics, version 18.0 (SPSS Inc., Chicago, IL, USA) and R software, version 3.4.1 (www.R-project.org). The threshold for statistical significance was a two-sided *p* < 0.05.

## Results

### Patient demographic data, clinical characteristics and molecular subtypes

The baseline characteristics of the patients are shown in Table 1. There were no differences between the training and validation cohorts in terms of demographic or clinical characteristics (*p* = 0.053–1.000).

In total, we included 73 (69.5%) patients with MGMT promoter methylation and 32 (30.5%) patients without MGMT promoter methylation. No significant difference was shown for the MGMT methylation status distribution in the training and validation cohorts (*p* = 0.156).

### Feature stability and reproducibility estimation

The statistical results of feature numbers after stability and reproducibility analysis are shown in Online Supplemental Fig. 2. Features extracted from tumour habitats exhibited statistically better performance than those extracted from peritumoral oedema habitats (reproducibility, *p* < 0.001; stability, *p* < 0.001).

### Single radiomics signatures formula and evaluation

The selected features and integration formulas for single radiomics signatures derived from each sequence and habitat are shown in Online Supplemental Table 1 and Appendix E5, respectively. A detailed explanation of each selected feature is shown in Online Supplemental Table 2. Single radiomics signatures from T1-WI-tumour, T1-WI-oedema, T2-FLAIR-tumour, and T2-FLAIR-oedema were verified as eligible radiomics signatures with AUCs > 0.7 in both the training and validation cohorts (Table 3). These four single radiomics signatures all showed significant differences (*p* < 0.05) in the MGMT methylated and unmethylated groups in both the training and the validation cohorts. Boxplots describing the distribution of the four radiomics signatures in the MGMT methylated and unmethylated groups are shown in Online Supplemental Fig. 3. However, neither ADC-tumour nor ADC-oedema performed with satisfactory results for MGMT identification. ADC-tumour did not perform well in the validation cohort (AUC = 0.590) while ADC-oedema did not perform well in the training cohort (AUC = 0.678). Detailed predictive indicators (AUC, accuracy, sensitivity and specificity) of each single radiomics signature are shown in Table 3.

**Table 3:** Diagnostic performance of single radiomics signatures, fusion radiomics signature, clinical factors and ADC values

Models	Training cohort N=74				Validation cohort N=31			
	Sensitivity	Specificity	Accuracy	AUC (95% CI)	Sensitivity	Specificity	Accuracy	AUC (95% CI)
Tumour T1	0.789	0.691	0.716	0.706 (0.567–0.845)	0.615	0.667	0.645	0.739 (0.558–0.921)
Tumour T2	0.947	0.782	0.824	0.916 (0.852–0.979)	0.538	0.889	0.742	0.701 (0.494–0.908)
Tumour ADC	0.895	0.655	0.716	0.815 (0.714–0.917)	0.692	0.389	0.516	0.590 (0.381–0.799)
Oedema T1	0.632	0.782	0.743	0.738 (0.601–0.875)	0.615	0.667	0.645	0.709 (0.519–0.900)
Oedema T2	0.684	0.727	0.716	0.778 (0.654–0.902)	0.615	0.778	0.710	0.752 (0.567–0.937)
Oedema ADC	0.632	0.782	0.743	0.678 (0.534–0.822)	0.615	0.889	0.774	0.816 (0.667–0.965)
Fusion radiomics	0.872	0.842	0.865	0.925 (0.861–0.989)	0.944	0.539	0.774	0.902 (0.785–1.000)
Clinical factors	0.737	0.600	0.6351	0.668 (0.548–0.789)	0.692	0.556	0.613	0.624 (0.448–0.800)
ADC values	0.632	0.691	0.676	0.649 (0.511–0.787)	0.615	0.722	0.677	0.603 (0.382–0.823)

*95% CI* 95% confidence interval, *AUC* area under curve, *T1* contrast-enhanced T1-weighted sequence, *T2* T2-FLAIR sequence

### Fusion radiomics signature formula and evaluation

The fusion radiomics signature combining the four single radiomics signatures was constructed with a Rad-score calculated as follows:

$$ {\displaystyle \begin{array}{c} Rad\hbox{-} score(fusion)=- 6.785- 1.026\ast {Signature}_{Oedema-T1}+ 3.950\ast {Signature}_{Oedema-T2}+\\ {} 2.907\ast {{Signature_{Tumo}}_{ur}}_{-T1}+{5.427}^{\ast }\ {Singature}_{Tumo ur-T2}\end{array}} $$

The optimum cut-off value of the fusion radiomics was 1.077 as per the Youden index. Patients were divided into predicted MGMT methylated (Rad-score ≥ 1.077) and unmethylated groups (Rad-score < 1.077) based on fusion Rad-scores.

**Fig. 3:**
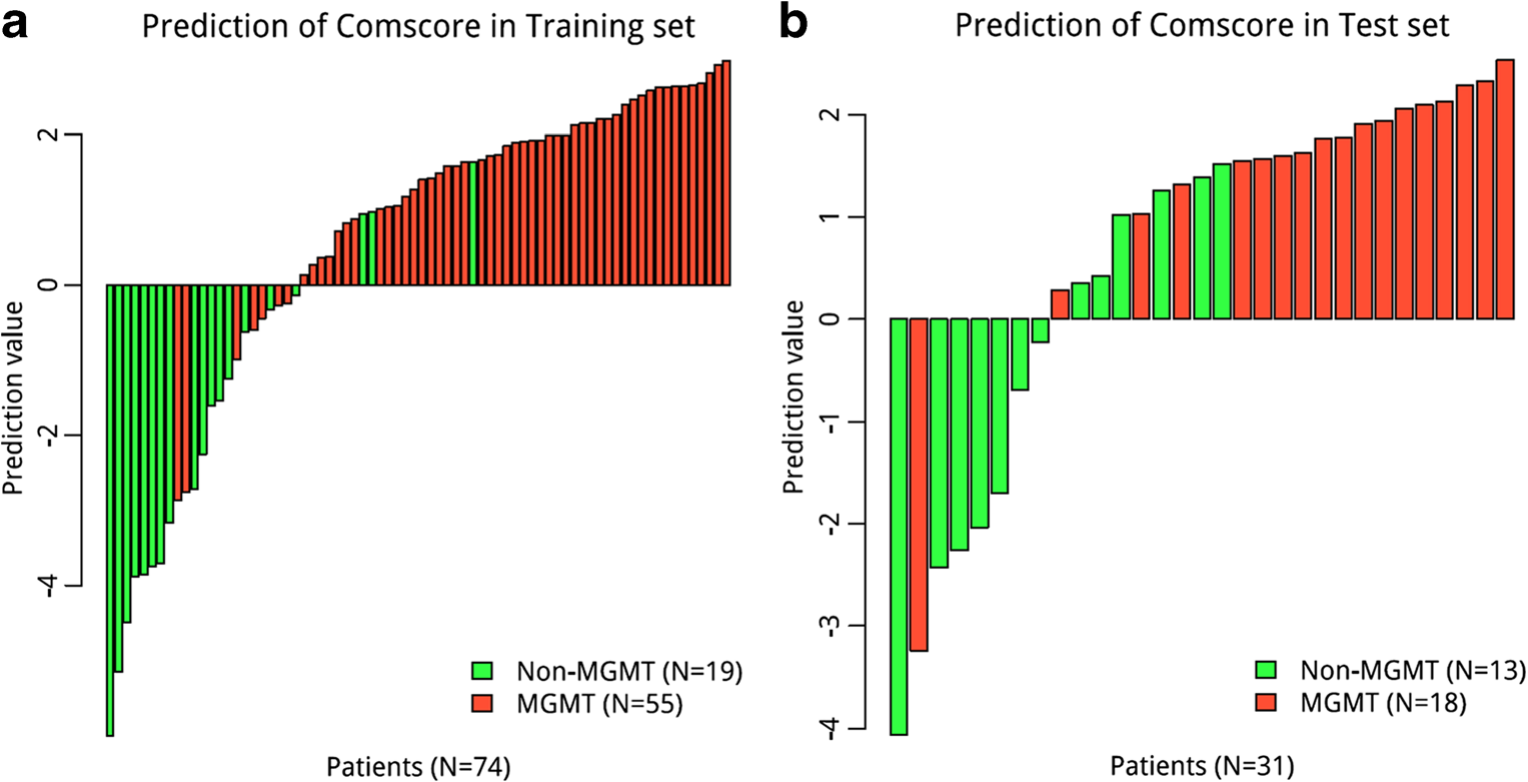
Barplots depicting the classification performance of the fusion radiomics signature. The red bar with a prediction value > 0 indicates that the signature successfully classifies the MGMT methylation patients; the red bar with a prediction value < 0 indicates that the signature fails to classify the MGMT methylation patients. For the green bar, the contrary applies

Barplots depicting the classification performance of the fusion signature in the training and validation cohorts are shown in Fig. 3. The fusion radiomics signature achieved optimal AUC values of 0.925 and 0.902 in the training and validation cohorts, respectively. Detailed predictive indicators (accuracy, sensitivity and specificity) of the fusion radiomics signature are shown in Table 3.

The fusion radiomics signature also had an outstanding performance in the stratification analysis, considering age, gender and grade (Table 4). It is noteworthy that the proposed radiomics signature does not require a priori knowledge of grading information because it behaved well for distinguishing MGMT methylation status not only in a grade II-IV cohort, but also in grade II, III and IV astrocytomas, separately.

**Table 4:** Stratification analysis of the fusion radiomics signature on training and validation cohorts

Subgroups			Fusion radiomics signature, median (IQR)						
			Training cohort			Validation cohort			
		MGMT (+)	MGMT (-)	AUC (95% CI)	*p*	MGMT (+)	MGMT (-)	AUC (95% CI)	*p*
Age, y	≤52	3.024 (2.215–3.706)	0.846 (-0.692–1.769)	0.918 (0.8149–1)	0.0022^*^	2.859 (2.681–3.135)	0.381 (-0.791–1.885)	0.9135 (0.775–1)	0.0012^*^
	>52	2.798 (1.500–3.136)	-2.088 (-2.777–0.461)	0.9447 (0.8649–1)	< 0.001^*^	2.626 (2.108–3.364)	1.177 (-0.675–1.950)	0.92 (0.7361–1)	0.052
Gender	Male	2.98 (2.219–3.371)	-1.899 (-2.752–0.218)	0.9469 (0.8835–1)	<0.001^*^	2.554 (1.922–3.025)	1.177 (-0.373–2.28)	0.7625 (0.5136–1)	0.067
	Female	2.741 (1.93–3.281)	-0.53 (-2.088–0.751)	0.9802 (0.7849–1)	<0.001^*^	2.938 (2.722–3.316)	-0.957 (-1.071–0.236)	1 (1–1)	0.0069^*^
Grade	II	2.991 (2.215–3.555)	0.444 (-0.46–0.942)	0.9417 (0.8473–1)	<0.001^*^	3.135 (2.747–3.309)	0.905 (0.046–1.657)	0.9643 (0.8653–1)	0.012^*^
	III	2.689 (1.992–3.265)	-2.383 (-3.119–-0.692)	0.9091 (0.7708–1)	<0.001^*^	2.859 (2.626–3.017)	1.500 (-0.625–2.1)	0.8444 (0.6224–1)	0.042^*^
	IV	2.798 (0.83–3.072)	–1.578 (-2.739–-0.264)	0.9444 (0.8227–1)	0.0028^*^	2.554 (2.477–2.632)	-0.165 (-1.227–1.257)	0.875 (0.5285–1)	0.27
—	—	2.807 (2.026–3.309)	-1.174 (-2.727–0.598)	0.9254 (0.8611–0.9896)	<0.001^*^	2.853 (2.631–3.162)	0.855 (-0.958–2.100)	0.9017 (0.7853–1)	<0.001^*^

*MGMT(+)*patients with oxygen 6-methylguanine-DNA methyltransferase (MGMT) methylation, *MGMT(-)* patients without MGMT methylation, *IQR* interquartile range

*p*-value < 0.05 indicates significant difference in the median radiomics score between the MGMT(+) and MGMT(-) groups

^*^p < 0.05

### Clinical model and ADC parameter evaluation

Only oedema degree was significantly different between the MGMT methylated and unmethylated groups in the training cohort (*p* = 0.0015). The AUCs of this clinical factor were 0.668 and 0.624 in the training and validation cohorts, respectively. The ADC parameter achieved AUC values of 0.649 and 0.603 in the training and validation cohorts, respectively. Detailed predictive indicators (sensitivity and specificity) of the clinical model and ADC parameter are shown in Table 3.

### Performance comparison

The fusion radiomics signature achieved the highest AUC among the three models. The Delong test showed a significant difference between the fusion radiomics signature and the clinical model (*p* = 0.008 and 0.011), and between the fusion radiomics signature and ADC parameter (*p* = 0.003 and 0.027) in the training and validation cohorts, respectively.

### Combined model construction and evaluation

During the combined model construction process, the AIC value was minimum when only taking the fusion radiomics signature into account. The AIC values were 48.79, 49.79, 51.25 and 53.11 when subsequently adding oedema degree, ADC of tumour area and ADC of oedema area to the fusion radiomics signature. Notwithstanding, we calculated the AUC of the combined model integrating all factors and the result was concordant with the AIC value; the combined model achieved an AUC of 0.921 in the training cohort and 0.868 in the validation cohort, which were slightly lower than those for the fusion radiomics signature alone.

### Prognostic value of the fusion radiomics signature

**Fig. 4:**
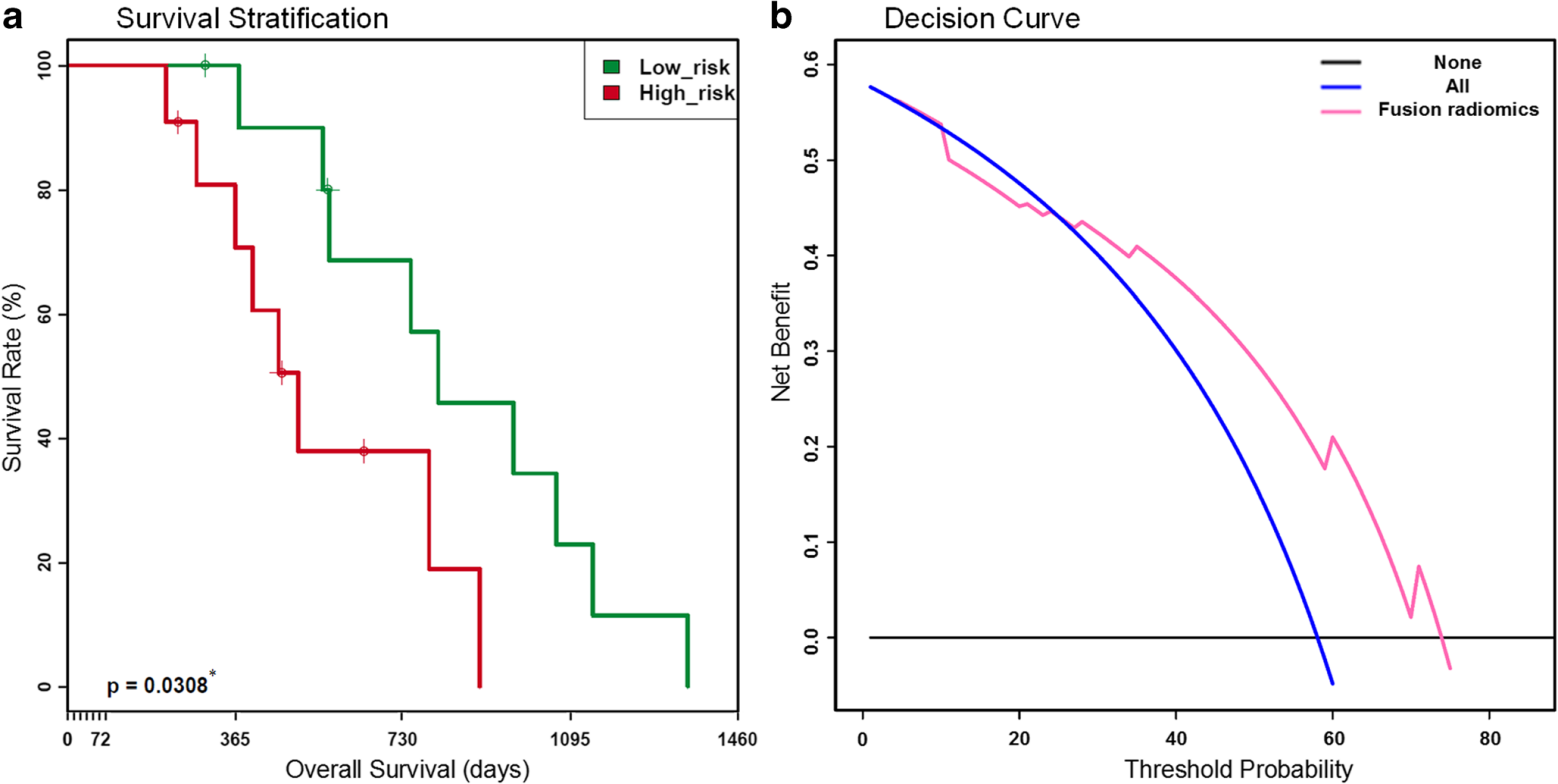
(**a**) Kaplan-Meier curve verifying the prognostic value of the fusion radiomics signature. Patients were successfully divided into high-risk (red line) and low-risk (green line) groups (*p* = 0.0308). (**b**) Decision curve analysis for the fusion radiomics signature on the overall cohort. The y-axis represents the net benefit and the x-axis represents the threshold probability. The threshold probability of the decision curve is 26% and the corresponding net benefit is 0.441. * *p* < 0.05

The fusion radiomics signature successfully divided patients treated with adjuvant TMZ chemotherapy into high-risk and low-risk OS groups with p = 0.03 (Fig. 4a). Moreover, the DCA showed that the fusion radiomics signature performed with higher net benefit (net benefit = 0.441) compared to simple stratification assuming that no patients had MGMT methylation or all patients had MGMT methylation (Fig. 4b).

## Discussion

In this study, a preoperative and low-cost radiomics analysis was used to integrate imaging features from tumour and peritumoral oedema habitats on CE-T1-WI and T2-FLAIR images to predict MGMT promoter methylation in patients with grade II-IV astrocytoma. Moreover, we verified the prognostic value of the fusion radiomics signature for patients who underwent resection followed by adjuvant TMZ chemotherapy.

Compared to clinical and conventional radiological factors [23, 24], our proposed radiomics signature exhibited excellent prediction performance. Additionally, the fusion radiomics signature integrating synergistic information outperformed each single radiomics signature from a simple habitat or sequence. Potential reasons for this observation are as follows: first, the fusion signature included more comprehensive information reflecting granular textural differences in the microenvironments and took in important archetypal imaging characteristics associated with MGMT methylation. Previous literature supports the value of the multi-habitat radiomics for predicting survival in patients with GBM [25–27]. Second, most of the effective features extracted in our study were textural features from Gabor transformation images, which conducted noise removal and filtration. Thus, these transformed images more effectively captured key tumour heterogeneity [25]. These findings agree with the radiomics hypothesis that gene phenotypic information of the tumour is reflected in radiological images [28, 29].

In previous work, Xi et al. showed that a radiomics signature derived from T1-WI, T2-WI and enhanced T1-WI was a potential imaging marker for the prediction of MGMT promoter methylation in GBMs, with prediction accuracy of 86.59% in the training cohort and 80% in the validation cohort [30]. However, MGMT methylated patients not only behaved well in GBM, but also presented with prolonged survival in lower-grade astrocytomas [4, 31]. Our study used an expanded cohort that included grade II-IV astrocytomas, and performed with superior AUCs of 0.926 and 0.902 in the training and validation datasets, respectively. Notably, our proposed fusion radiomics signature has the power to distinguish MGMT methylation in separate grade II, III and IV (GBM) cohorts, as well as in a grade II-IV cohort. It can predict MGMT methylation status directly without the need for a pathological grading prerequisite. Considering that the most significant advantage of radiomics is its non-invasive characteristics, pre-knowledge of grading that requires biopsy critically limits the clinical application of radiomics, while our results showed great advances on the previous study with improved high accuracy. This finding will strongly promote radiomics application in clinical practice.

Furthermore, we also investigated whether a combined model integrating clinical factors, radiological factors and fusion radiomics signature would outperform the signature alone. However, adding oedema degree and ADC values to the fusion radiomics signature caused minor deterioration rather than improvement in prediction performance. This indicated that adding clinical and radiological factors to the radiomics signature increased the complexity without increasing the prediction accuracy. The oedema degree and ADC values, as kinds of semi-quantitative clinical and quantitative radiological factors, partially depended on the subjective judgement of radiologists (sometimes with strong reservations), and their prediction performance were poorer compared with the radiomics signature. Thus when incorporating these factors into the radiomics signature, there was no additional positive effect on the improvement of the prediction performance, but rather an increased complexity of the prediction model. Additionally, even though it was reported that ADC values were correlated with MGMT promoter methylation and prognosis in GBM [22, 32, 33], our results indicated that radiomic features extracted from T1-CE and T2-FLAIR sequences performed better than those extracted from the ADC sequence. A potential reason for this observation is the relatively poor imaging resolution of ADC, which limited the stability and robustness of the derived radiomics features.

MGMT promoter methylation has been shown to be associated with longer OS, [34]. In our study, MGMT promoter methylation status successfully stratified astrocytoma patients treated with adjuvant TMZ chemotherapy into two groups with significant prognostic differences, consistent with previous research [6]. We also validated the proposed fusion radiomics signature for assessing TMZ chemotherapy effect. Using the cut-off value of the fusion Rad-score, patients with positive radiomics scores after TMZ chemotherapy had significantly longer OS than patients with negative scores (*p* = 0.03), revealing another possible clinical application of this genetic prediction tool.

The present study had several limitations. First, our model was trained and validated using retrospective data collected from a single institution. A large-scale prospective and multicentre validation cohort collection is currently underway. Second, our radiomics analysis only predicted MGMT promoter methylation prediction from T1-CE, T2-FLAIR and ADC map images, which are the most common structural MR images. Additional scanning sequences such as dynamic susceptibility contrast, susceptibility-weighted imaging and diffusional kurtosis imaging will be included in future studies to further improve predictive performance. Third, the relationship between imaging features and critical molecular markers such as IDH and 1p19q should also be studied in future research. Finally, the manual segmentation method used to delineate ROIs in this study (tumour and oedema) was quite time consuming. Semi-automatic or deep learning-based automatic segmentation methods may enhance the objectivity of our method and promote the seamless integration of this technology into clinical application.

In conclusion, habitat-based MRI radiomics could provide a non-invasive imaging strategy for the preoperative prediction of MGMT promoter methylation in patients with grade II–IV astrocytomas, and has prognostic implications for TMZ chemotherapy, which may serve as a tool for guiding individualised treatment decision making.

## Electronic supplementary material

ESM 1 (DOCX 865 kb)

## Abbreviations

ADC: Apparent diffusion coefficient
AUC: Area under the curve
DCA: Decision curve analysis
GBM: Glioblastoma
IDH: Isocitrate dehydrogenase
MGMT: Oxygen 6-methylguanine-DNA methyltransferase
MRI: Magnetic resonance imaging
OS: Overall survival
ROC: Receiver operating characteristic
ROI: Region of interest
T1-WI: T1-weighted imaging
T2-FLAIR: T2-weighted fluid-attenuated inversion recovery images
TMZ: Temozolomide
