# Supplementary material for: A multi-sequence and habitat-based MRI radiomics signature for preoperative prediction of MGMT promoter methylation in astrocytomas with prognostic implication
Source: Eur Radiol. 2018 Jul 23;29(2):877–88. doi: 10.1007/s00330-018-5575-z (PMC6302873; doi:10.1007/s00330-018-5575-z)
Supplement: Supplementary file 1 — (DOCX 865 kb) [file 330_2018_5575_MOESM1_ESM.docx]

**Supplemental Figures**

**Figure 1**

**
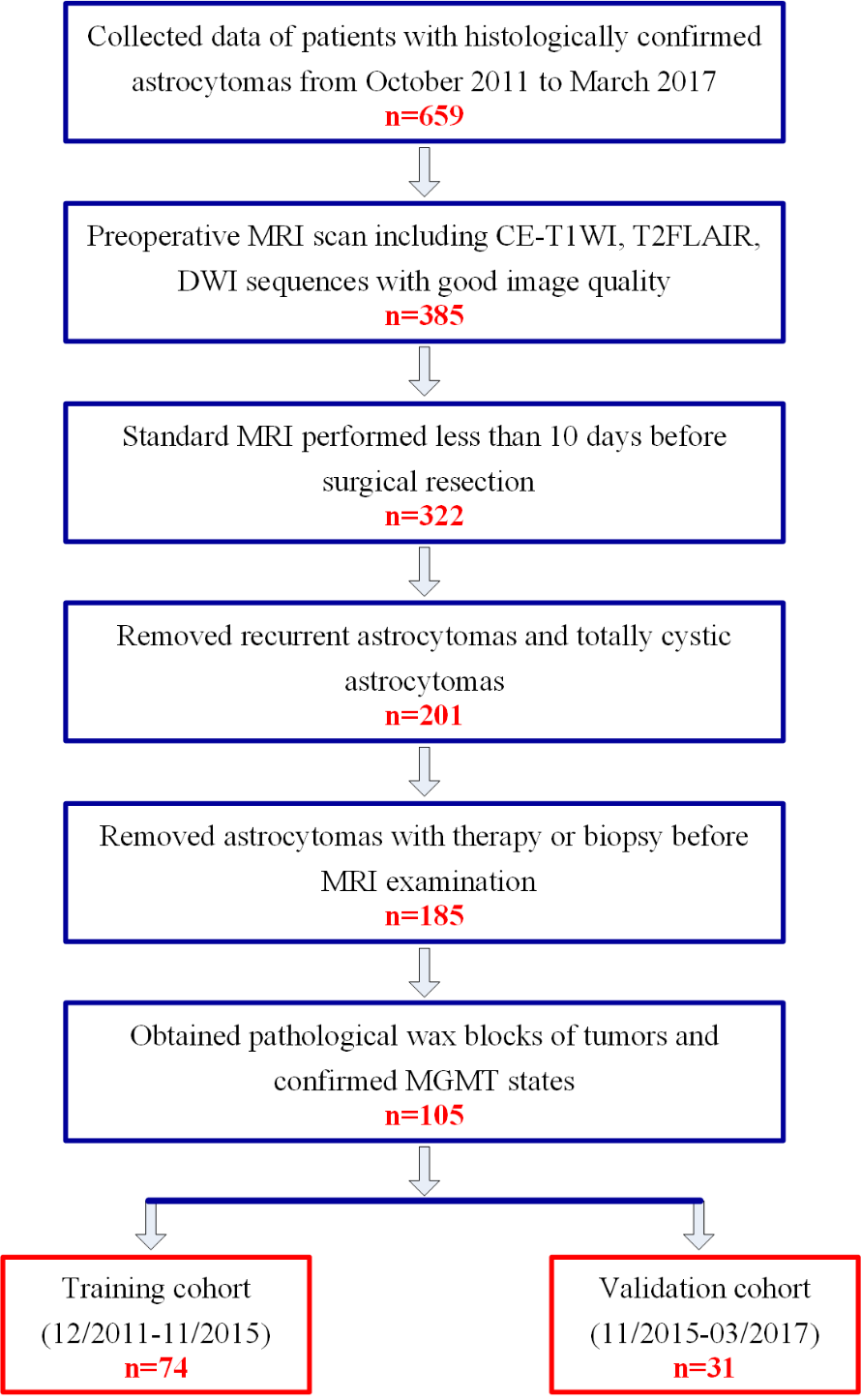
**

**Figure 1:** Patient recruitment pathway and training/validation cohorts division for the radiomics analysis.

**Figure 2**


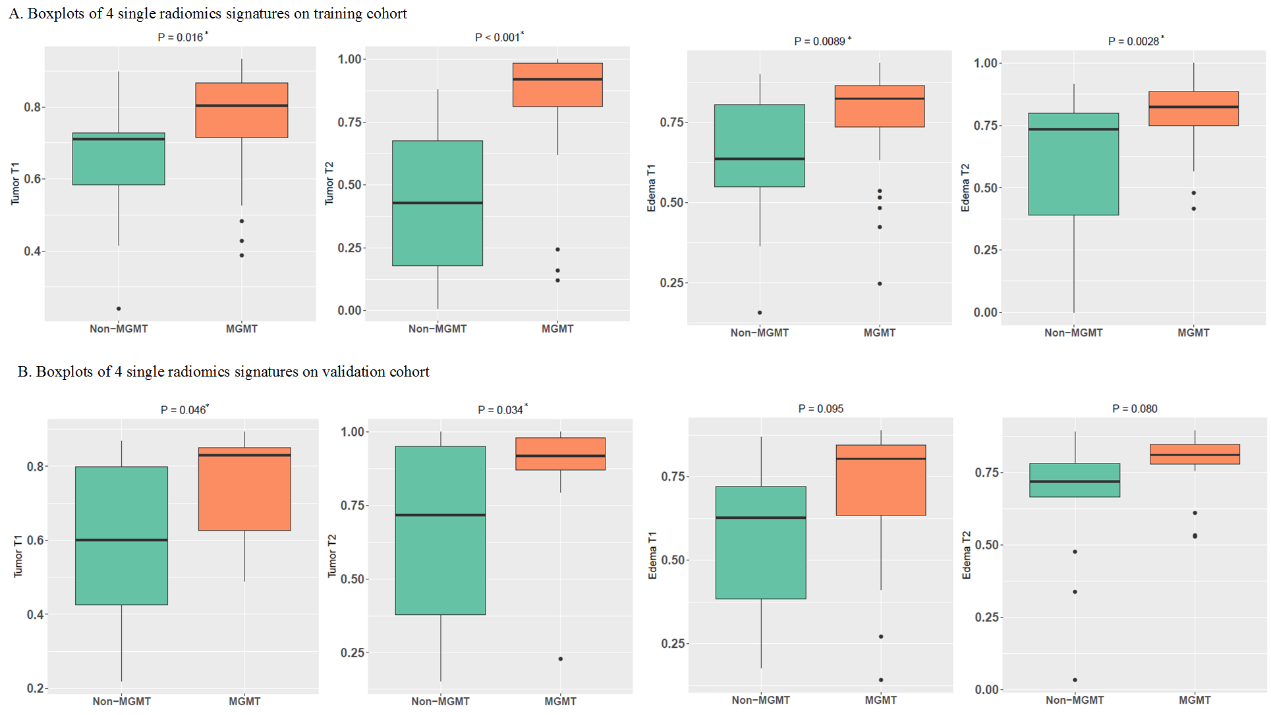


**Figure 2:** Features with CCC > 0.75 and ICC > 0.80 were selected into further analysis. We can see from the figure that features extracted from tumor habitats performed more reproducible and stable than those extracted on peritumoral edema habitat (ICC: p < 0.001; CCC: p < 0.001). ^*^P < 0.05.

**Figure 3**


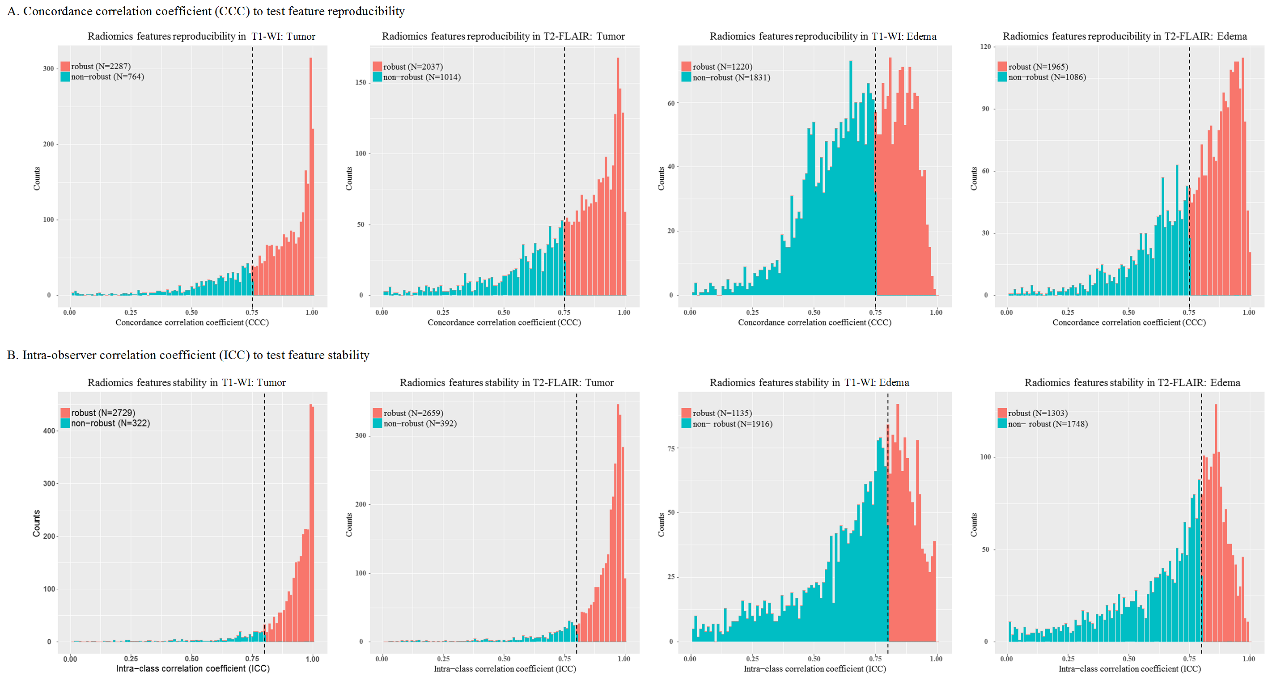


**Figure 3:** Boxplots presented the distribution of each single radiomics signature scores between MGMT methylated and unmethylated groups on training and validation cohorts, respectively. P (on the top of each subfigure) less than 0.05 represents for significant difference between the MGMT methylated and unmethylated groups. ^*^P < 0.05.

**Supplemental Tables**

**Table 1. The selected features for each single radiomics signature on different habitats and sequences**

| **Habitats and sequences** | **Radiomics features** | | | | |
| --- | --- | --- | --- | --- | --- |
| **Tumor T1** | Tumor_T1_  gabor28_fos_range |  |  |  |  |
| **Tumor T2** | Tumor_T2_  gabor35_glcm_energy | Tumor_T2_  ori_fos_range | Tumor_T2_  ori_glszm_ZSNU | Tumor_T2_  gabor34_fos_minimum | Tumor_T2_  gabor27_glszm_LGLZE |
| **Tumor ADC** | Tumor_ADC_  gabor2_glszm_LZHGE | Tumor_ADC_  gabor28_fos_maximum |  |  |  |
| **Edema T1** | Edema_T1_  gabor17_glcm_energy | Edema_T1_  gabor37_fos_maximum |  |  |  |
| **Edema T2** | Edema_T2_  R5W5_fos_median | Edema_T2_  gabor34_glszm_GLNU |  |  |  |
| **Edema ADC** | Edema_ADC_  gabor38_glrlm_LRHGLE |  |  |  |  |

**Note:** Features are selected by Bayesian information criteria after maximum relevance and minimum redundancy algorithm. T1: contrast-enhanced T1-weighted sequence; T2: T2-FLAIR sequence.

**Table 2. Detailed information of the selected features in T1-Tumor, T2-Tumor, T1-Edema and T2-Edema signature construction.**

| **Feature name** | **Formula** | **Content** |
| --- | --- | --- |
| Tumor_T1_gabor28_fos_range | fos_range  = The range intensity value of **X** | The range intensity value of the first order statistical histogram of the Tumor_T1 image transformed by Garbor 28 filter |
| Tumor_T2_gabor35_glcm_energy | glcm_energy  =$\sum_{i=1}^{N_{\text{g}}} \sum_{j=1}^{N_{g}} \left[C(i,j)\right]^{2}$ | The energy of the Gray-Level Co-Occurrence Matrix of the Tumor_T2 image transformed by Garbor 35 filter |
| Tumor_T2_ori_fos_range | The range intensity value of **X** | The range intensity value of the first order statistical histogram of the Tumor_T2 image |
| Tumor_T2_ori_glszm_ZSNU | glszm_ZSNU  =$\sum_{i=1}^{N_{g}} \left[\sum_{j=1}^{N_{z}} Z(i,j)\right]^{2}$ | Zone Size Non-uniformity of Gray-level size zone matrix of the Tumor_T2 image |
| Tumor_T2_gabor34_fos_minimum | The minimum intensity value of **X** | The minimum intensity value of the first order statistical histogram of the Tumor_T2 image transformed by Garbor 34 filter |
| Tumor_T2_gabor27_glszm_LGLZE | glszm_LGLZE  = $\frac{\sum_{i=1}^{N_{\text{g}}} \left[\sum_{j=1}^{N_{z}} \frac{Z(i,j)}{i^{2}}\right]}{\sum_{i=1}^{N_{g}} \sum_{j=1}^{N_{z}} Z(i,j)}$ | Low Gray Level Zone Emphasis of Gray-level size zone matrix of the Tumor_T2 image transformed by Garbor 27 filter |
| Tumor_ADC_gabor2_glszm_LZHGE | Glszm_LZHGE  =$\frac{\sum_{i=1}^{N_{g}} \left[\sum_{j=1}^{N_{z}} i^{2}\cdot j^{2}\cdot Z\left(i,j\right)\right]}{\sum_{i=1}^{N_{g}} \sum_{j=1}^{N_{z}} Z\left(i,j\right)}$ | Long Zone High Gray-Level Emphasis of Gray-level size zone matrix of the Tumor_ADC image transformed by Gabor 2 filter |
| Tumor_ADC_gabor28_fos_maximum | The minimum intensity value of **X** | The minimum intensity value of the first order statistical histogram of the Tumor_ADC image transformed by Gabor 28 filter |
| Edema_T1_gabor17_glcm_energy | glcm_energy  = $\sum_{i=1}^{N_{\text{g}}} \sum_{j=1}^{N_{g}} \left[C(i,j)\right]^{2}$ | The energy of the Gray-Level Co-Occurrence Matrix of the Edema_T1 image transformed by Garbor 17 filter |
| Edema_T1_gabor37_fos_maximum | The maximum intensity value of **X** | The maximum intensity value of the first order statistical histogram of the Edema_T1 image transformed by Garbor 37 filter |
| Edema_T2_R5W5_fos_median | The median intensity value of **X** | The median intensity value of the first order statistical histogram of the Edema_T2 image transformed by Laws R5W5 filter |
| Edema_T2_  gabor34_glszm_GLNU | glszm_GLNU  = $\sum_{i=1}^{N_{g}} \left[\sum_{j=1}^{N_{z}} Z(i,j)\right]^{2}$ | Gray Level Non-uniformity of Gray-level size zone matrix of the Edema_T2 image transformed by Gabor 34 filter |
| Edema_ADC_gabor38_glrlm_LRHGLE | glrlm_LRHGLE= $\frac{\sum_{i=1}^{N_{g}} \left[\sum_{j=1}^{N_{r}} i^{2}\cdot j^{2}\cdot R(i,j\vert\theta)\right]}{\sum_{i=1}^{N_{g}} \sum_{j=1}^{N_{r}} R(i,j\vert\theta)}$  | Long Run High Gray Level Emphasis of gray-level run length matrix of Edema_ADC image transformed by Gabor 38 filter |

**Where:**

**X** be either the original image, the image transformed by 40-scale gabor filter or 15-scale laws filter.

$\bar{X}$ be median intensity value for image **X**

$R(i,j|\theta)$ be the value of row i and column j in Gray-Level Run-Length Matrix for a direction $\theta$

for image **X**

$C(i,j)$ be the value of row i and column j in Gray-Level Co-Occurrence Matrix for image **X**

$Z(i,j)$ be the value of row i and column j in Gray-Level Size Zone Matrix for image **X**

$N_{g}$ be the number of discrete intensity values in the image

$N_{r}$ be the number of different run lengths

$N_{z}$ be the size of the largest homogeneous region

$N_{p}$ be the number of voxels in the image

$\mu_{x}(i)$ be the mean of row i

$\mu_{y}(j)$ be the mean of column j

$\sigma_{x}(i)$ be the standard deviation of row i

$\sigma_{y}(j)$ be the standard deviation of column j

*Note:* Detailed image transformation, see supplement method: radiomics features.

**Table 3. Median ROIs on T1-WI, T2-FLAIR and ADC map .**

| **Habitat&**  **Sequence** | **Training cohort**  **N=74** | | | **Validation cohort**  **N=31** | | | **P (Inter)** |
| --- | --- | --- | --- | --- | --- | --- | --- |
|  | **MGMT(+)**  **(cm^2^)** | **MGMT (-)**  **(cm^2^)** | **P (Intra)** | **MGMT(+)**  **(cm^2^)** | **MGMT (-)**  **(cm^2^)** | **P (Intra)** |  |
| **Tumor T1** | 14.8391 | 14.7424 | 0.5532 | 20.3664 | 18.6698 | 0.6085 | 0.1637 |
| **Tumor T2** | 15.1752 | 14.7424 | 0.5899 | 18.9873 | 18.1618 | 0.3529 | 0.5011 |
| **Tumor ADC** | 15.2236 | 13.2621 | 0.5313 | 19.7402 | 18.1055 | 0.3118 | 0.4913 |
| **Edema T1** | 7.5844 | 12.1029 | 0.3474 | 9.5217 | 12.8084 | 0.0724 | 0.5767 |
| **Edema T2** | 8.0283 | 12.7754 | 0.3078 | 11.9809 | 13.5754 | 0.0579 | 0.6619 |
| **Edema ADC** | 9.4395 | 9.3779 | 0.7855 | 8.0552 | 10.3623 | 0.0728 | 0.9276 |

**Note:** MGMT(+) represents for patients with oxygen 6-methylguanine-DNA methyltransferase (MGMT) methylation; MGMT(-) represents for patients without MGMT methylation. P(Intra) is the result of uni-variable analyses between methylated and unmethylated groups. P(Inter) represents whether there exists significant difference between training and validation cohorts. Unless otherwise specified, data are numbers of patients, with percentages in parentheses.

**Supplemental Appendix**

**E1: Inclusion and exclusion criteria**

The inclusion criteria was as follows: (a) pathology diagnosed as astrocytoma with no previous history of primary central nervous system tumors; (b) astrocytoma grade II–IV; (reported with potential benefit from TMZ chemotherapy), (c) tissue wax block available for confirmation of MGMT methylation status; (d) preoperative standard MR imaging available, including contrast enhanced T1-weighted imaging (CE-T1-WI), T2-weighted fluid-attenuated inversion recovery (T2FLAIR) imaging, and diffusion weighted imaging (DWI); and (e) MR images acquired 10 days before surgical resection. The exclusion criteria were: (a) preoperative therapy including radiotherapy, chemotherapy, or chemoradiotherapy; (b) a history of biopsy before MRI acquisition; (c) recurrent astrocytomas; (d) totally cystic astrocytomas; and (e) inadequate MRI with motion artifacts or unclear images.

**E2: The PCR amplification and conditions**

PCR amplification was accomplished with DRR006 kit (Takara, Japan) using 40-μl reaction volume containing 1μl each forward and reverse primer, 4μl 10× buffer, 26.6μl sterile water, 3.2μl dNTPs, 0.2μl Taq polymerase, and 4μl bisulphite-treated DNA. The PCR conditions were as follows: 94°C for 2 min; 50 cycles of 94°C for 20 s, 55°C for 20 s, and 72°C for 20 s; and 72 °C for 5 min. A 25-μl volume of PCR product was subjected to pyrosequencing on a PyroMark Q96 (Qiagen, Germany) as per manufacturer instructions. Pyrosequencing yielded data for 10 CpG sites within the MGMT promoter and the percentage methylation obtained for each CpG was averaged across the 10 CpGs in PCR reactions. Astrocytomas were considered to be methylated if the average methylation was ≥ 8% (≥ mean ± 2 standard deviations for non-neoplastic brains), unmethylated cases had average methylation < 8% [1].

**E3: The acquisition protocol**

The acquisition protocol included CE-T1-WI (repetition time/echo time, 195 ms/4.76 ms; field of view, 240mm; thickness/slice interval, 5.0 mm/1.5 mm; and matrix, 256 × 256) and T2-FLAIR (repetition time/echo time, 8000 ms/95 ms; field of view, 240 mm; thickness/slice interval, 5.0 mm/1.5 mm; and matrix, 256 × 256). CE-T1-WI was obtained after the injection of 0.1 mmol/kg of gadolinium chelate contrast medium. Echo planar imaging (EPI) was used to perform DWI (repetition time/echo time, 3000 ms/87 ms; field of view, 240 mm; thickness/slice interval, 6 mm/1 mm; and matrix, 160 × 160). Implemented b-values of DWI were 0 and 1000 mm2/s. The DWI scan time was 45 s. The original DWI maps were transmitted to Advanced Workstation 4.4 to generate axial ADC maps using GE software processing.

**E4: Detailed description of radiomics process**

*ROI segmentation* *–* To ensure the accuracy of ROI delineation, manual segmentation was performed in a blinded fashion by 2 radiologists with 10 and 15 years of work experience, respectively, using the opening software ITK-SNAP (<http://www.radiantviewer.com>). We selected the final ROI as the overlap area of the segmentation results from both radiologists, and each ROI was validated by a senior radiologist with 20 years of experience. The tumor border and peritumoral edema were both delineated on the slice with maximum tumor area on each sequence. Segmentation standard as follows: (1) ROIs of unenhanced tumors in the lesion and peritumoral edema area were delineated according to transverse T2-FLAIR and DWI images. Tumor intensity was lower than peritumoral edema intensity on T2-FLAIR images and higher than peritumoral edema intensity on DWI. (2) ROIs of enhanced tumors in the lesion and peritumoral edema area were delineated according to transverse CE-T1-WI and T2-FLAIR images. (3) If the border between the tumor and peritumoral edema was not clear both on CE-T1-WI and T2-FLAIR images, DWI and ADC images were used to define the border. Tumor intensity was higher than peritumoral edema intensity on DWI images and lower than peritumoral edema intensity on ADC images. ROIs of tumor and peritumoral edema area on ADC maps were obtained with reference to CE-T1-WI, T2-FLAIR, and DWI images in accordance with the segmentation standard as previously described.

*Radiomic feature extraction* *–* We extracted 3051 features from CE-T1-WI and T2-FLAIR images and ADC maps on tumor and peritumoral edema habitats, respectively. The feature set was divided into 5 groups: (I) shape and size, (II) first order statistic, (III) texture, (IV) Gabor and (V) Laws features. Shape and size features changed the direct-viewing imaging characteristics into machine-read quantitative features, reflecting the morphologic traits of the tumor lesion. First order statistic features counts in the statistical indexes of the imaging histogram on the tumor lesion area. Texture features were achieved based on four textural matrixes: gray level co-occurrence matrix [2], gray level run-length matrix [3], gray level size zone matrix [4] and neighborhood gray-tone difference matrix [5]. Gabor and Laws features were extracted based on the images with Gabor or Laws transformation.

1. Gabor transformation

Gabor transformation is a short time Fourier transform and reflected the features from frequency domain, manifesting both the spatial and local imaging variation information. The kernel function of Gabor transform is as follows:

$$G\left( Z \right)=\frac{{K_{v}}^{2}}{\sigma^{2}}e^{-\frac{{K_{v}}^{2}*Z^{2}}{2\sigma^{2}}}\left( e^{i*K*Z}-e^{-\frac{\sigma^{2}}{2}} \right)$$

$$K_{v}=K_{max}/(f^{V})$$

$$K=K_{v}e^{i\frac{\pi*U}{8}}$$

Where σ is a constant with value ofπ; K_max_ is the maximum sampling frequency with value ofπ/2; f is the sampling step length in the frequency domain with value of $\sqrt{2}$; V is the frequency index with the value range of [0, 1, 2, 3, 4] and U is the direction index with the value range of [0, 1, 2, 3, 4, 5, 6, 7]. Thus, through the combination of V and U, the original image could be transformed into 40 scale domains. We extracted first order statistic, texture features after the Gabor transformation.

1. Laws transformation

Laws transformation is implemented through capturing 5 kinds of subtle textures including level, edge, spot, wave and ripple. These five basic texture patterns is depicted based on 5 one-dimensional kernels:

L1 = [1/16 4/16 6/16 4/16 1/16]; (level)

L2 = [-1 -2 0 2 1]; (edge)

L3 = [-1 0 2 0 -1]; (spot)

L4 = [-1 2 0 -2 1]; (wave)

L5 = [1 -4 6 -4 1]; (ripple)

The two-dimensional kernel was achieved through the following calculation:

$${Kernel}_{i,j}={(L}_{i}^{'}*L_{j}+L_{j}^{'}*L_{i})/2 \forall i,j=1,2,\cdots,5$$

Let the original image convolve with the corresponding 15 two-dimensional kernal_i,j_, we could achieve the laws filtered images. First order statistic features were extracted from the resulting laws filtered images.

Types and names of the features are shown in Table 6. All the features extraction was implemented using Matlab 2014a (MathWorks, Natick, MA, USA).

**Table 6. Radiomics features used in this experiment.**

| **Non-texture Features (25)** | |
| --- | --- |
| - **Shape and Size Features (8)** | |
| **Reference** | **Feature** |
| —— | Compactness1  Compactness2  Maximum 3D diameter  Spherical disproportion  Sphericity  Surface area  Surface to volume ratio  Volume |
| - **First Order Statistic Features (17)** | |
| **Reference** | **Feature** |
| —— | Energy  entropy  Kurtosis  Maximum  Mean  Mean absolute deviation  Median  Minimum  Range  Root mean square  Skewness  Standard deviation  Sum  Uniformity  Variance  Entropy after normalization  Uniformity after normalization |
| **Textural Features (75)** | |
| - **GLCM Features (22)** | |
| **Reference** | **Feature** |
| Haralick et al 1973 [2] | Autocorrelation  Cluster prominence  Cluster shade  Cluster tendency  Contrast  Correlation  Difference entropy  Dissimilarity  Energy  Entropy  Homogeneity1  Homogeneity2  Information measure of correlation 1  Information measure of correlation 2  Inverse difference moment normalized  Inverse difference nomalized  Inverse variance  Maximum probability  Sum average  Sum entropy  Sum variance  Covariance |
| - **GLRLM Features (11)** | |
| **Reference** | **Feature** |
| Galloway 1975 [3] | Short Run Emphasis (SRE)  Long Run Emphasis (LRE)  Gray Level Non-Uniformity (GLN)  Run Length Non-Uniformity (RLN)  Run Percentage (RP) |
| Chu et al 1990 [6] | Low Gray Level Run Emphasis (LGLRE)  High Gray Level Run Emphasis (HGLRE) |
| Dasarathy and Holder 1991 [7] | Short Run Low Gray Level Emphasis (SRLGLE)  Short Run High Gray Level Emphasis (SRHGLE)  Long Run Low Gray Level Emphasis (LRLGLE)  Long Run High Gray Level Emphasis (LRHGLE) |
| - **GLSZM Features (13)** | |
| **Reference** | **Feature** |
| Galloway 1975 [3] | Small Zone Emphasis (SZE)  Large Zone Emphasis (LZE)  Gray-Level Nonuniformity (GLN)  Zone-Size Nonuniformity (ZSN)  Zone Percentage (ZP) |
| Chu et al 1990 [6] | Low Gray-Level Zone Emphasis (LGZE)  High Gray-Level Zone Emphasis (HGLZE) |
| Dasarathy and Holder 1991 [7] | Small Zone Low Gray-Level Emphasis (SZLGE)  Small Zone High Gray-Level Emphasis (SZHGE)  Large Zone Low Gray-Level Emphasis (LZLGE)  Large Zone High Gray-Level Emphasis (LZHGE) |
| Thibault et al 2009 [4] | Gray-Level Variance (GLV)  Zone-Size Variance (ZSV) |
| - **NGTDM Features (5)** | |
| **Reference** | **Feature** |
| Amadasun and King 1989 [5] | Coarseness  Contrast  Busyness  Complexity  Strength |

*Stability and reproducibility analysis –* The stability (inter-observer consistency) and reproducibility (test-retest reliability) of radiomic features were analyzed in 20 randomly selected images. Intra-class correlation coefficients (ICCs) and concordance correlation coefficients (CCCs) were calculated to measure feature variability. Only stable features with ICCs > 0.80 and CCCs > 0.75 were qualified. We further performed dynamic range (DR) analysis to select distinguishable features [8, 9]. Features with DR > 0.9 were selected for the final feature pool [9].

*Feature selection and radiomics signature construction* *–* To reduce the number of irrelevant and redundant radiomic features, Mann-Whitney U test was primarily used to assess the statistical power of features for distinguishing between MGMT methylated and unmethylated groups in the training dataset. Features with p < 0.05 were retained as potential predictive features. Then, we ranked the selected potential features based on minimum redundancy and maximum relevance score [10]. Features were finally selected to build the radiomics signature using logistic regression model with the Bayesian information criterion as the stopping rule [11]. This process was performed on the 3 MR sequences and 2 habitats to yield 6 single radiomics signatures. A fusion radiomics signature was constructed combining better behaved single radiomics signatures (AUC > 0.7 in both the training and validation cohorts) using logistic regression model.

**Appendix E5: Single radiomics signature formula:**

CE-T1-WI_Tumor:

*Signature_Tumor-T1_ = 1/ (1+exp (-1*(2.949760089 -0.003265396* Tumor_T1_gabor28_fos_range)))*

CE-T1-WI_Edema:

*Signature_Edema-T1_ = 1/ (1+exp (-1*(4.542903730 -10.521015276*Edema _T1 _gabor17_glcm_energy -0.006437571* Edema _T1 _gabor37_fos_maximum)))*

T2-FLAIR_Tumor:

*Singature_Tumor-T2_ = 1/ (1+exp (-1*(-3.002646112 +129.494523339* Tumor_T2_gabor35_glcm_energy +0.005468715*Tumor_T2_ori_fos_range -0.012151107*Tumor_T2_ori_glszm_GLNU -0.020111834*Tumor_T2_gabor34_fos_minimum +193.546251339* Tumor_T2_gabor27_glszm_LGLZE)))*

T2-FLAIR_Edema:

*Signature_Edema_T2_ = 1/ (1+exp (-1*(2.59504994 -1.32197178* Edema _T2_R5W5_fos_median -0.05376349* Edema _T2_ gabor34_glszm_ZSNU)))*

ADC-Map_Tumor:

*Signature_Tumor_ADC_ = 1/ (1+exp (-1*(-3.8889614867+0.0003443383*Tumor_ADC_ gabor2_glszm_LZHGE + 0.0030500250*Tumor_ADC_ gabor28_fos_maximum)))*

ADC-Map_Edema:

*Signature_Edema_ADC_ = 1/ (1+exp (-1*(1.7525029325 -0.0009672214* Edema_ADC_*

*gabor38_glrlm_LRHGLE)))*

**Reference**

1. Reifenberger G, Hentschel B, Felsberg J, et al. (2012) Predictive impact of MGMT promoter methylation in glioblastoma of the elderly. *International Journal of Cancer.* 131(6): 1342-1350.

2. Haralick R M, Shanmugam K, Dinstein I. (1973) Textural Features for Image Classification. *Systems Man & Cybernetics IEEE Transactions on.* smc-3(6):610-621.

3. Galloway M M. (1975) Texture analysis using gray level run lengths. *Computer Graphics & Image Processing.* 4(2):172-179.

4. Thibault G, Fertil B, Navarro C, et al. (2009) Texture Indexes and Gray Level Size Zone Matrix Application to Cell Nuclei Classification. *In Pattern Recognition and Information Processing (PRIP).*

5. Amadasun, M. and R. King. (1989) Textural features corresponding to textural properties. *Systems Man & Cybernetics IEEE Transactions on.* 19(5): p. 1264-1274.

6. Bocchino, C., et al. (1990) Use of gray value distribution of run lengths for texture analysis. *Pattern Recognition Letters.* 11(6): p. 415-419.

7. Dasarathy, B.V. and E.B. Holder. (1991) Image characterizations based on joint gray level—run length distributions. Pattern Recognition Letters. 12(8): p. 497-502.

8. Landis JR and Koch GG. (1977) The measurement of observer agreement for categorical data. *Biometrics.* 33(1): 159-174.

9. Balagurunathan Y, Gu Y, Wang H, et al. (2014) Reproducibility and prognosis of quantitative features extracted from CT images. *Translational Oncology.* 7(1): 72-87.

10. Peng H, Long F and Ding C. (2005) Feature selection based on mutual information criteria of max-dependency, max-relevance, and min-redundancy. *IEEE Transactions on pattern analysis and machine intelligence*. 27(8): 1226-1238.

11. Weakliem DL (1999)A critique of the Bayesian information criterion for model selection. *Sociological Methods & Research*. 27(3): 359-397.
